# Parthenolide inhibits ERK and AP-1 which are dysregulated and contribute to excessive IL-8 expression and secretion in cystic fibrosis cells

**DOI:** 10.1186/1476-9255-8-26

**Published:** 2011-10-12

**Authors:** Aicha Saadane, Jean Eastman, Melvin Berger, Tracey L Bonfield

**Affiliations:** 1 Department of Pediatrics, Case Western Reserve University, 11100 Euclid Avenue, BRB-822 Cleveland Ohio 44106, OH 44106, USA

## Abstract

**Background:**

Excessive secretion of IL-8 characterizes cystic fibrosis (CF). This has been attributed to excessive activation of epithelial cell I-κB Kinase and/or NFκB. Maximum IL-8 production requires 3 cooperative mechanisms: 1) release of the promoter from repression; 2) activation of transcription by NFκB and AP-1; 3) stabilization of mRNA by p38-MAPK. Little is known about regulation of IL-8 by MAPKs or AP-1 in CF.

**Methods:**

We studied our hypothesis *in vitro *using 3-cellular models. Two of these models are transformed cell lines with defective versus normal cystic fibrosis transmembrane conductance regulator (CFTR) expression: an antisense/sense transfected cell line and the patient derived IB3-1/S9. In the third series of studies, we studied primary necropsy human tracheal epithelial cells treated with an inhibitor of CFTR function. All cell lines were pretreated with parthenolide and then stimulated with TNFα and/or IL-1β.

**Results:**

In response to stimulation with TNFα and/or IL-1β, IL-8 production and mRNA expression was greater in CF-type cells than in non-CF controls. This was associated with enhanced phosphorylation of p38, ERK1/2 and JNK and increased activation of AP-1. Since we previously showed that parthenolide inhibits excessive IL-8 production by CF cells, we evaluated its effects on MAPK and AP-1 activation and showed that parthenolide inhibited ERK and AP-1 activation. Using a luciferase promoter assay, our studies showed that parthenolide decreased activation of the IL-8 promoter in CF cells stimulated with TNFα/IL-1β.

**Conclusions:**

In addition to NFκB MAPKs ERK, JNK and p38 and the transcription factor AP-1 are also dysregulated in CF epithelial cells. Parthenolide inhibited both NFκB and MAPK/AP-1 pathways contributing to the inhibition of IL-8 production.

## Introduction

Cystic fibrosis (CF) is characterized by repeated and progressive airways infection, inflammation, and obstruction. It is now clear that the immune and inflammatory responses in CF lung are disproportionate to the threat posed by infection [1-6]. Bronchoalveolar lavage (BAL) fluid from patients with CF contains higher concentrations of IL-8 and PMN than BAL from patients without CF but with similar burdens of bacteria or LPS [7-9]. Considerable evidence indicates that activation of NFκB is prolonged and excessive in epithelial cell lines, mice and humans with defective CFTR expression or function [5,6,10-14]. NFκB clearly plays a key role in the regulation of expression of pro-inflammatory cytokines, chemokines and mucins which are important in CF. However, despite its major role; it is unlikely that NFκB alone should be sufficient for maximal transcriptional activation or induction of all the genes that are involved in this complex disease. Several studies suggest that expression of IL-8 is subject to multiple and coordinated regulation by mitogen activated protein kinases (MAPKs) and AP-1, in addition to NFκB [12,15-17]. Holtmann and colleagues have proposed a model in which the extent of IL-8 production is the net result of 3 regulatory mechanisms: 1) release of the promoter from repression; 2) transcriptional activation by NFκB and AP-1; and 3) stabilization of mRNA by p38 MAPK [16]. IL-8 gene expression is also regulated in part through remodeling of chromatin structure which is under the control of various changes including histone acetylation and DNA methylation [18-20]. Recently, we showed that parthenolide, a naturally occurring sesquiterpene lactone from the medicinal plant feverfew, inhibits IκB kinase (IKK), NFκB activation and IL-8 secretion by CF epithelial cells [14].

Very few studies focused on MAPKs activity [12,15] but none of these showed the prolonged activation of the MAPKs that could explain the prolonged and unresolved inflammatory responses documented in CF patient. Moreover, to our knowledge no study has shown excessive or prolonged activation of AP-1 in CF.

In the present study, we investigated the following two hypotheses: 1) To determine if the signaling pathway going through the MAPKs: p38, extracellular-regulated protein kinase (ERK), and Jun-N terminal protein kinase (JNK) to the transcription factor activator-protein-1 (AP-1) signaling is dysregulated in CF epithelial cells; 2) To determine whether this pathway can be manipulated by parthenolide. To study MAPKs and AP-1 involvement in CF epithelial IL-8 production we used 3 different CF epithelial cell models and their corresponding controls. The first model is the human bronchial epithelial cell line16 HBE, stably transfected with antisense oligonucleotides, inhibiting the expression of CFTR (AS). The control for this cell line is 16HBE cells stably transfected with sense oligonucleotides (S). The second model uses cells obtained from a patient with CF, this is designated IB3 and the control cell line which has been corrected with full-length CFTR, this is designated S9 [22]. The third model involved using human primary tracheal epithelial cells treated with CFTR inhibitor 172 (CFTR_inh_172) [21].

## Methods

### Cell culture

We used two different sets of cell lines with CF defects and their corresponding controls (Table 1). The first was 16 HBE human bronchial epithelial cell line stably transfected with an anti-sense (AS) oligonucleotide which inhibits expression of CFTR; and a sister cell line transfected with CFTR sense oligonucleotide, (S) as the control. These have been described previously [21] and were kindly provided by Dr. Pamela Davis (Case Western Reserve University, Cleveland). The second was IB3-1 cells from a patient with CF, and the control cell line, S9 cells, which was rescued from CFTR deficiency by stable transfection with full-length functional CFTR [22]. Cells were maintained in a 5% CO_2 _incubator at 37°C using MEM (Mediatech, Inc. Herndon, VA) for AS and S cell lines and LHC-8 media (Biosource, Camarillo, CA) for IB3-1 and S9 cell lines. All media contained penicillin/streptomycin and 10% fetal bovine serum.

**Table 1:** Cell Lines

CF	Wild Type
AS (16HBE stably transfected with an anti-sense oligonucleotide)	S (16HBE stably transfected with CFTR sense oligonucleotide)

IB3-1 (CF patient)	S9 (CF patient-transfected with full length CFTR)

HTE+CFTR_inh_172 (necropsy human tracheal epithelial treated with CFTR inhibitor 172)	HTE+DMSO (necropsy human tracheal epithelial treated with DMSO)

We also used human tracheal epithelial cells (HTE) recovered from necropsy specimens, as previously described [21]. Cells were grown in an air-liquid interface (ALI) on collagen-coated, semi-permeable membrane as previously described. Cells were allowed to differentiate for 3 to 4 weeks, then switched to liquid-liquid interface, (LLI) and treated with either DMSO 1:1000 (vehicle control) or 20 μM CFTR_inh_172 [21]. Drugs were added to both the apical and basolateral sides and refreshed every 24 hours. After 72 hours, cells were pretreated with 15 μM parthenolide or vehicle alone for 1 hour before treatment with TNFα at 100 ng/ml for 3 h. The HTE cells were all from the same donor specimens. Six filters were used for DMSO and six with CFTR_inh_172.

### Experimental conditions

Cell lines were plated at 2 × 10^6 ^cells/well on vitrogen-coated 6-well plates. Twenty-four hours after plating, the cells were switched to serum-free medium for 18 h. IL-8 production was induced by treating AS and S cells with TNFα (100 ng/ml, Sigma St. Louis, MO) with or without IL-1β (100 ng/ml, Sigma St. Louis, MO), and IB3-1 and S9 with TNFα at 30 ng/ml [2,14]. Viability and possible cytotoxicity of the cytokines were determined by trypan blue exclusion [14]. As in our previous studies, cell lines were pretreated with 40 μM parthenolide for AS and S and 15 μM for IB3-1 and S9 (Sigma, St. Louis, MO) [14] for 1 h before treatment with TNFα and/or IL-1β. Parthenolide was dissolved in dimethylsulfoxide (DMSO), such that the final maximum concentration of DMSO in the experimental media was 0.04%. Controls contained the same concentration of DMSO. At various times, media were harvested and assayed for IL-8 (R&D Systems, Minneapolis, MN). Unstimulated controls were run in each experiment. After removal of media, the epithelial cell monolayers were washed with ice cold PBS and harvested by scraping, then pelleted at 600 × g for 5 min at 4°C. Separate cytosolic and nuclear proteins extracts were prepared according to manufacturer's instructions (BioVision Research products, Mountain View, CA). Extracts were then used for analysis of NFκB and AP-1. In another set of experiment cells were lysed using PhosphoSafe (Novagen, Madison, WI) containing protease and phosphatase inhibitors. Protein concentrations were determined using the Bradford method (Bio-Rad Laboratories) and data for IL-8 production were expressed as pg/mg cellular protein.

### RNA isolation, reverse transcription and real-time PCR

RNA was extracted using Trizol or the RNeasy^® ^Mini kit (Qiagen). For reverse transcription, 5 μg total RNA was adjusted to 8 μl. One μl of oligo (dt) (0.5 μg/μl) and 1 μl dNTP mix (10 mM each) were added. The mixtures were denatured at 65°C for 5 min and chilled on ice. cDNA was produced by adding a mixture containing 200 mM Tris-HCl, pH 8.4, 500 mM KCl), MgCl2, DTT, 1 μl RNase OUT, and 1 μl Superscript ™ II reverse transcriptase. The samples were incubated at 42°C for 50 min and at 70°C for 15 min then chilled on ice. RNase H (1 ul) was added, followed by incubation at 37°C for 20 min. The cDNA solution was subsequently diluted 20 times with water and stored at -80°C. Primers were designed using Primer Express software (Applied Biosystems, Foster City, CA). Transcript levels were normalized using the housekeeping gene GAPDH. We have previously determined that GAPDH expression is constitutive in the cell lines and was not s not altered by TNF-α or IL-1β treatment (data not shown). Samples were run in triplicate on an ABI Prism 7700 sequence detector (Applied Biosystems, Foster City, CA) according to the manufacturer's instructions for 40 cycles, and the average threshold cycle (Ct) was determined. Changes in IL-8 mRNA levels were expressed as ΔCt and ΔΔCt and the expression level of IL-8 gene was represented as fold increase: 2^-ΔΔCT^, where ΔΔCt = [ΔCt_sample stimulated_)] - [ΔCt _sample unstimulated_] and ΔCt = [Ct_sample_]- [Ct_GAPDH_].

### Electrophoretic Mobility Shift Assay (EMSA)

Aliquots of nuclear extracts from cell lines (5 μg) were suspended in binding buffer [10 mM Tris-HCl, pH 7.5, 1 mM MgCl2, 50 mM NaCl, 0.5 mM EDTA, 0.5 mM DTT, 4% glycerol, 0.5 μg poly (dI-dC)] at room temperature for 10 min then incubated for an additional 20 min with ^32^P-radiolabeled consensus oligonucleotides: 5'AGTTGAGGGGACTTTCCCAGGC-3' for NFkB assays 5'-CGCTTGATGAGTCAGCCGGAA-3' for AP-1 (both from Promega, Madison, WI). Protein binding of the oligonucleotides was analyzed using 6% non-denatured PAGE and autoradiography [14]. Cold competitors were used to assure the specificity of binding of each oligonucleotide. A Panomics Luminex based kit was also used to study NFκB and AP-1 activation, according to manufacturer's instructions (Panomics Fremont, CA).

### Immunoblotting for MAP kinases: ERK, JNK and p38

Aliquots of cell extracts (15 μg protein) were separated by 10% SDS-PAGE and transferred onto nitrocellulose membrane. The western blots were probed using rabbit polyclonal antibodies specific for phosphorylated and total p38, JNK and ERK (Cell Signaling, Inc., Beverly MA) and immunoreactive proteins were visualized by enhanced chemiluminescence [5,6,14]. Blots were also probed with monoclonal anti-β-actin to assure equal protein loading.

### Transfection and luciferase promoter assays

AS and S cell lines were cotransfected with firefly luciferase construct dependent on the IL-8 promoter, and a reporter plasmid mediating constitutive expression of *Renilla *luciferase as a transfection control [23]. Cells were lysed for measurement of firefly and *Renilla *luciferase activities using reagents from Promega as previously described. Firefly luciferase activity was normalized by comparison to the *Renilla *luciferase activity in the same sample.

### Statistical Analysis

For cytokine data, 0 was assigned to values below the limit of detection of the ELISA assays. All data are expressed as mean ± SEM. Data were compared using the student's t-test. Comparisons between CF and wild type with/without stimulations, 95% confidence interval, significance p ≥ 0.05. Data is product of 3 to 4 different experiments with n = 4 to 8 per each time point and condition.

## Results

### Loss of CFTR Results in high accumulation of IL-8 mRNA

**Figure 1:**
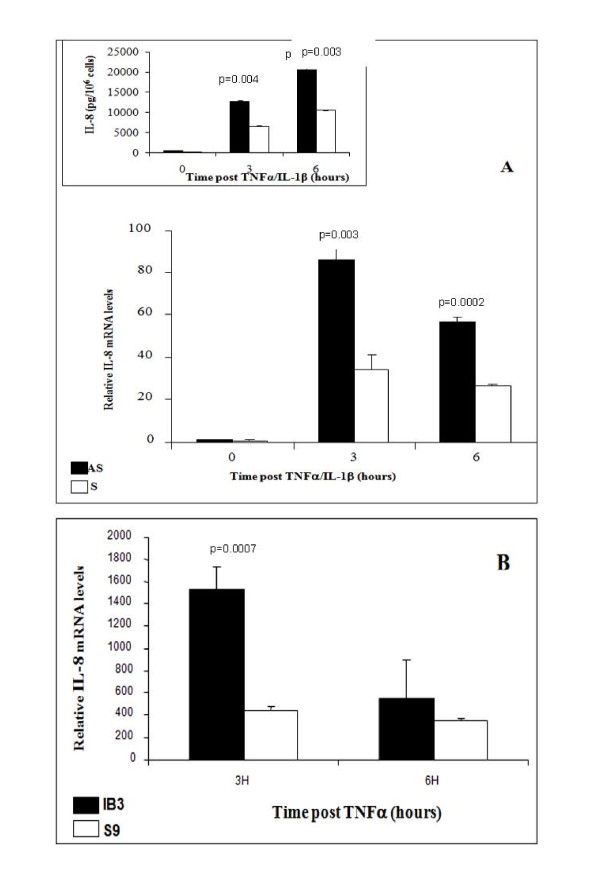
**IL-8 expression and production by "CF like" like (Solid bars)-and "wild-type like" cells (open bars) upon stimulation with IL-1β and/or TNFα**. IL-8 mRNA expression was evaluated by real time-PCR. (A) IL-8 mRNA were significantly higher in AS cells at 3 and 6 h (p = 0.003 and p = 0.0002 respectively) and (B) in IB3-1 cells at 3 h (p = 0.0007). The media were also collected and subject to ELISA to determine IL-8 production. Similarly to the content of IL-8 mRNA, IL-8 protein were significantly high in AS cells at 3 and 6 h (p = 0.004 and p = 0.003 respectively) compared to S cells (A, inset).

CF cell lines stimulated by TNFα/IL-1β secreted much greater concentrations of IL-8 than similarly stimulated non-CF cells. We previously reported that this was associated with increased and persistent activation of IKK and NFκB in the AS cells as compared to S cells [2,14]. In this study, we determined the expression of IL-8 mRNA in AS/S and IB3-1/S9 cell lines in response to IL-1β and/or TNFα by real time PCR. The mRNA contents were normalized for GAPDH and the results are given in relative units. Following treatment with TNFα/IL-1β, IL-8 mRNA was significantly higher in AS cells compared to S cells (Figure 1A). At 3 h post stimulation, and in accordance with the high production of IL-8 protein (Figure 1 inset), IL-8 mRNA was increased by more than 85 fold in AS cells (p = 0.003), while in S cells the increase was only 33 fold (Figure 1A) when compared to their non-stimulated baseline controls. At 6 h after stimulation, IL-8 mRNA was still elevated by 56 fold in the AS cells (p = 0.0002), as compared to 26-fold in the S cells when compared to their non-stimulated baseline controls. Similarly IB3-1 cells also increased IL-8 mRNA accumulation much more than S9 cells (Figure 1B). At 3 h post stimulation IL-8 mRNA was increased by more than 1500 fold in IB3-1 cells (p = 0.0007) while in S9 cells the increase was only 400 fold, when compared to their non-stimulated baseline controls. At 6 h after stimulation IL-8 mRNA was still elevated by 600 fold in the IB3-1 cells as compared to 350 fold in the S9 cells compared to baseline controls.

### TNF-α and/or IL-1β causes increased activation of AP-1 in CF as compared to control cells

**Figure 2:**
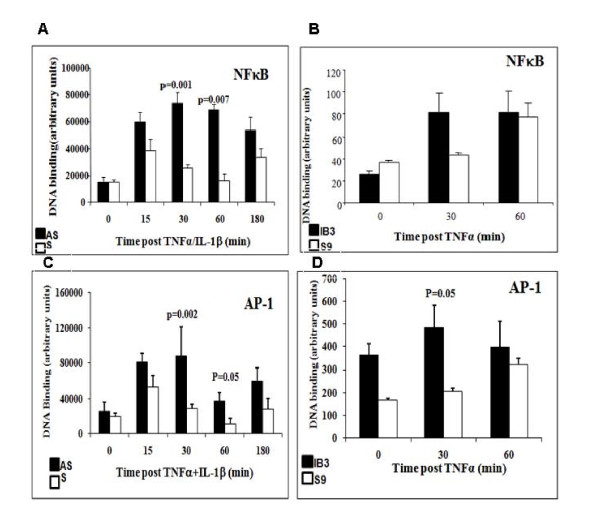
**Loss of CFTR Results in high activation of the transcription factors NFκB and AP-1**. AS and S cells were incubated in serum free media for 18 h then were stimulated with TNFα/IL-1β for the indicated time. Subsequently, nuclear extracts were prepared then analyzed for NFκB (A, B) and AP-1 (C, D). Cells not stimulated with TNFα/IL-1β are shown as 0 time. A and C; The amount of complexed NFκB and AP-1 probe respectively estimated by image scanning and expressed in arbitrary units. Results are shown as mean ± SEM of three separate experiments (n = 5-6 for each time point and condition). Solid bars, AS cells and open bars S cells. NFκB. AP-1 activation was significantly increased in AS cells at 15 and 30 min after TNFα/IL-1β stimulation (p = 0.003 and p = 0.0002, respectively) compared to S cells.

Recently, there have been major advances in understanding how different signaling pathways coordinately regulate IL-8 transcription and mRNA stabilization in response to external stimuli. Besides its binding sites for NFκB, the IL-8 promoter also contains binding sites for activator protein-1 (AP-1), which is not essential for baseline expression but is required for maximal expression of IL-8 [16,17,24,25]. To understand how the two transcription factors might contribute to the tremendous increase in IL-8 protein secretion by CF epithelial cells, we studied the activation of AP-1 as well as NFκB. As reported previously, and shown here as a positive control, unstimulated AS and S cells have little detectable active nuclear NFκB (Figure 2A, time 0). However, stimulation with TNFα/IL-1β increased binding of NFκB by 15 min in both cell types (Figure 2A, B). In AS cells, the increase in NFκB activation was significantly higher at 30 and 60 min (p = 0.001 and p = 0.007, respectively). The same phenomenon was observed with the IB3 and S9 model. TNFα stimulation significantly increased NFκB binding at 30 minutes (Figure 2B). However, the NFκB levels of activation were comparable between the IB3 (CF cells) and S9 (controls) at 60 minute, potentially related to the different origins of the cell lines. The AS/S and IB3/S9 models from the same studies were also evaluated for AP-1 activation post-TNFα stimulation. Baseline AS/S and IB3/S9 cells had little or no nuclear AP-1 (Figure 2C and 2D, time 0). Incubation with TNFα/IL-1β increased AP-1 activation significantly in both AS (p = 0.002) and IB3 (p = 0.05) cell lines by 30 min Similarly, the elevated levels of AP-1 in AS cells were sustained out to 180 minutes consistent with the NFκB studies, with the IB3 comparable with the S9 cells (Figures 2C and 2D). These data suggest that both NFκB and AP-1 activation is dysregulated in CF epithelial cells, potentially contributing to the excessive IL-8 message expression and protein secretion observed under these conditions.

### TNFα- and/or IL-1β-induces over-activation of p38, ERK and JNK in cells with defective CFTR expression

**Figure 3:**
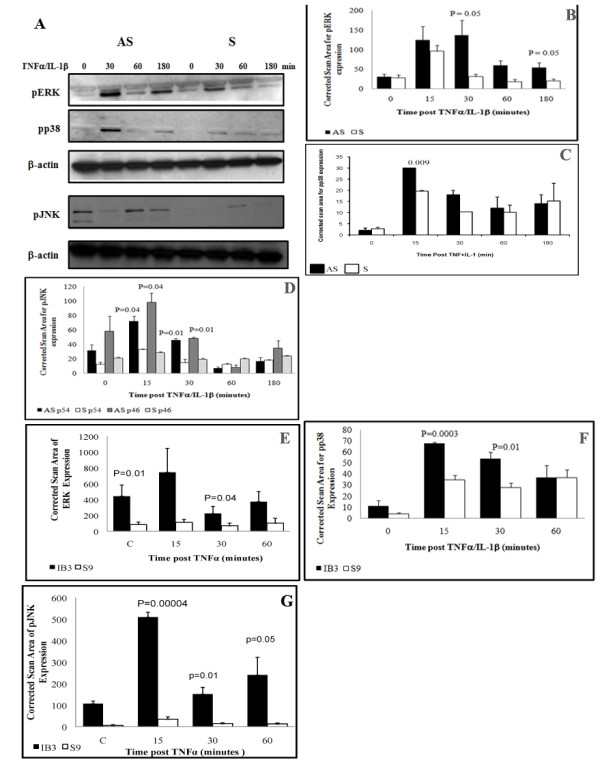
**Activation of MAPKs in IL-1β and/or TNFα stimulated AS/S and IB3-1/S9 cells**. Both pair of cell lines were incubated in serum free media for 18 h then stimulated with IL-1β and/or TNFα for the indicated time points. At the indicated time media was removed and cells washed then total cell extracts were prepared which serve to perform western blot to detected phosphorylation and total protein of the MAPKs ERK, p38 and JNK in AS and S cells (A, B, C, D, respectively), and IB3-1 and S9 cells (E, F, G, respectively). Summary graph of data for MAPKs phosphorylation determined by image analysis of densitometry from autoradiographs. Data show mean ± SEM of scan area of pERK, pp38 and pJNK (n = 5 to 8 in each time point and condition) corrected for scan area of β-actin in the same gel lane.

Because AP-1 is activated by mitogen-activated protein kinases (MAPKs) [17,26,27], we sought to determine whether activation of these enzymes might be associated with AP-1 activation in the CF cells. MAPK Phospho-p38, JUN-N terminal protein kinase (JNK), and the extracellular-regulated protein kinase (ERK) cascades are active by phosphorylation through dual specificity MAPK kinases (MKK) and may all contribute to IL-8 expression. We found no difference in baseline ERK-phosphorylation between b AS and S cell lines (time 0, Figure 3A). TNFα/IL-1β stimulation of AS cells resulted in ERK phosphorylation at all time points. ERK phosphorylation was significantly increased in AS cells at 30 and 180 min (p = 0.05)) (Figure 3B, solid bars), while it was markedly reduced by 30 min and barely detectable at 60 and 180 min in the S cells (Figure 3B, open bars). Figure 3A shows marked increases in phosphorylated p38 at in the AS cells after 15 and 30 minutes post-stimulation with TNFα/IL-1β, which was only modest in the S cells. A similar pattern was observed for the phosphorylation of the p54 and p46 isoform of JNK (Figures 3A and 3C). Phospho-p54 was significantly increased in AS cells at 15 and 30 min (p = 0.04 and p = 0.01, respectively) (Figure 3C, solid pars). Phospho-p46 was also significantly high in AS cells at 15 and 30 min (p = 0.04 and p = 0.01, respectively) as compared to control S cells (Figure 3C, hatched bars). In all cases TNFα/IL-1β stimulation did not alter the total amounts of these proteins (data not shown) suggesting that the treatment change activity of the MAPKs rather than the abundance of the proteins. In comparison to AS cells, all 3 MAPKs were significantly elevated in IB3-1 cells. Figure 3D shows that ERK phosphorylation is very significant at the baseline in IB3-1 cells (time 0, p = 0.01) compared to S9 cells. TNFα stimulation significantly increased phospho-ERK in IB3-1 at 30 min (p = 0.04) (Figure 3D, solid bars). Phospho-p38 was significantly increased in TNFα stimulated IB3-1 cells at 15 and 30 min (Figure 3E, p = 0.003 and p = 0.01, respectively) (Figure 3E, solid bars). Figure 3F, shows a significant elevation in phospho-JNK at all-time point in IB3-1 cells at 15, 30 and 60 min (p = 0.00004, p = 0.01 and p = 0.05 respectively) compared to control S9 cells.

### Parthenolide inhibits TNFα/IL-1β-induced activation/nuclear translocation of AP-1

**Figure 4:**
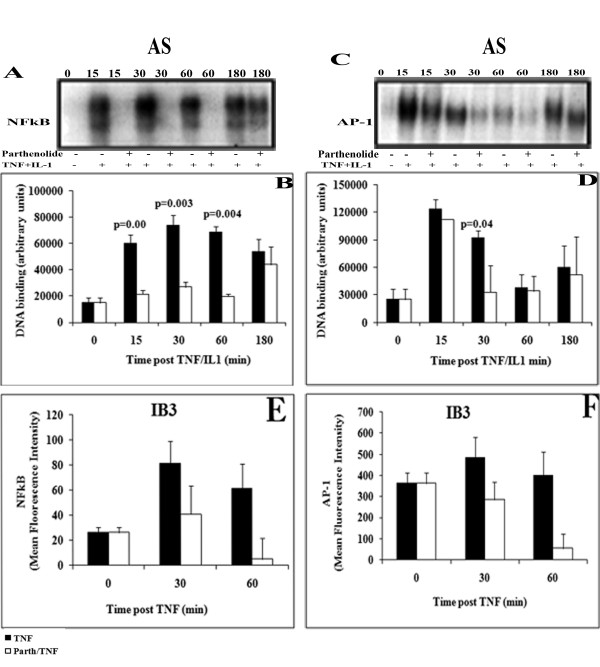
**AS cells were pretreated with parthenolide or vehicle (placebo) for 1 h and then stimulated with TNFα/IL-1β for the indicated time**. Nuclear extracts were prepared and then analyzed by EMSA for both transcription factors NFκB and AP-1. Representative autoradiograph of EMSA for (A) NFκB activation and (C) for AP-1 were presented. Summary graph of data for NFκB (B) and AP-1 (D) activation determined by densitometry from 4 time course experiments (n = 5 in each time point and condition), mean ± SEM are shown. Solid bar, placebo; and open bars, parthenolide. Parthenolide pretreatment inhibited the activation of (B) NFκB at 15, 30 and 60 min p = 0.003, p = 0.003 and p = 0.004, respectively), and (D) AP-1 at 30 min (p = 0.004).

Parthenolide is an anti-inflammatory drug thought to be able to suppress inflammation through inhibiting NFκB, amongst other effects [14]. The observed differences in MAPKs activity opened new questions about the mechanisms of parthenolide's effects in CF cells. Therefore, we sought to determine if the inhibitory activities of parthenolide also extended to the MAPK-AP-1 pathway. AS and S cells were pretreated with parthenolide then stimulated with TNFα/IL-1β as in Figure 1 and 2. Nuclear extracts were prepared and DNA-binding activity of AP-1 was measured by EMSA. For comparison, NFκB was also assayed. The inhibition of NFκB activation by parthenolide was similar to what we reported previously [14]. Parthenolide pretreatment inhibits significantly NFκB activation in AS cells at 15, 30 and 60 min (p = 0.003, p = 0.003 and p = 0.004, respectively) (Figure 4A and 4B). Pretreatment with parthenolide also inhibits significantly AP-1 activation in AS at 30 min (p = 0.04) (Figure 4C and 4D). Densitometry of the parthenolide inhibitor effect on NFκB and AP-1 activity in IB3-cells with (white bars) and without (solid bars) parthenolide is shown (Figures 4E and 4F).

### Parthenolide inhibited ERK1/2 and JNK phosphorylation and stabilized p38 phosphorylation

**Figure 5:**
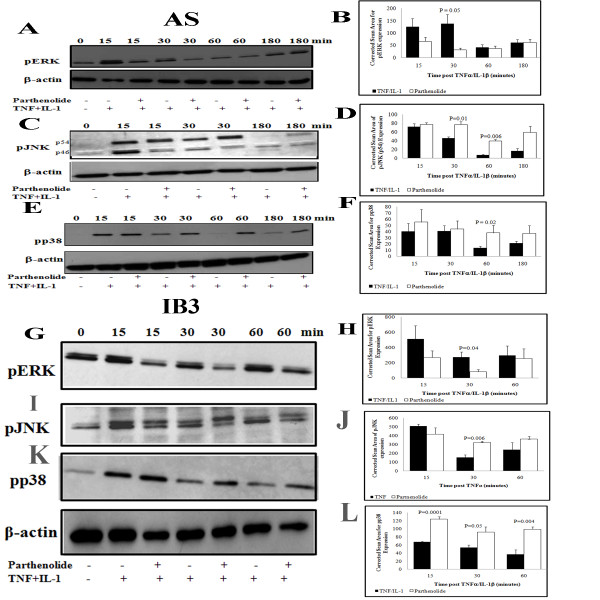
**AS and IB3-1 cells were pretreated with parthenolide or vehicle (Placebo) for 1 h and then stimulated with IL-1β and/or TNFα for the indicated time**. Total cells protein extracts were prepared then analyzed for phosphorylated MAPK pERK, MAPK pJNK and the MAPK pp38 in AS cells (A/B, C/D and E/F, respectively) and in IB3-1 cells (G/H, I/J and K/L respectively). Summary graph of data for phosphorylated MAPKs determined by image analysis of densitometry from autoradiograph. Data show mean ± SEM of scan area of MAPK ERK, JNK and p38 expression (n = 5 to 8 for each time point and condition) corrected for scan area of β-actin in the same gel lane. In both cell lines Parthenolide inhibited cytokine-induced phosphorylation of ERK, but stabilized the phosphorylation of JNK and p38. Solid bar, placebo; open bar, parthenolide-treated cells.

We hypothesized that parthenolide's inhibition of AP-1 activation might be due to inhibition of the upstream MAPKs which activate this transcription factor. Because the p38 MAPK pathway is also believed to regulate a specific, post-transcriptional step in IL-8 mRNA processing, we focused on determining if parthenolide alters p38-phosphorylation [16,17]. Since MAPKs ERK and JNK activate AP-1, we also evaluated the effect of parthenolide on their activation. Figures 5A and 5B show that pretreatment with parthenolide inhibited phosphorylation and activation of ERK 1 and 2 at 15 and 30 min and the inhibition was significant at 30 min (p = 0.05). In contrast, parthenolide stabilized the phosphorylation of JNK, especially p46 at 30 and 60 min (p = 0.01 and p = 0.006, respectively) (Figure 5C and 5D, open bars), and the phosphorylated of JNK- p54 was not significantly different after parthenolide pretreatment (data not shown). Also, pre-treatment with parthenolide significantly stabilized phosphorylated p38 at 60 min (p = 0.02) (Figure 5E and 5F). Parthenolide pretreatment did not alter the total amount of all three proteins in the cells (data not shown).

In IB3-1 cells stimulated with TNFα alone, parthenolide pretreatment has the same effect as in AS cells stimulated with TNFα/IL-1β. Parthenolide pretreatment significantly inhibited phosphorylation and activation of ERK 1 and 2 at 30 min (p = 0.04) (Figure 5G and 5H, open bars), however pre-treatment with parthenolide stabilized phosphorylated phospho-JNK at 30 min (p = 0.006) (Figure 5I and 5J, open bars), and also stabilized the phosphorylated p38 at all time points 15, 30 and 60 min after TNFα stimulation (p = 0.0001, p = 0.05 and p = 0.004, respectively) (Figure 5K and 5L, open bars).

### Effects of parthenolide on IL-8 mRNA

**Figure 6:**
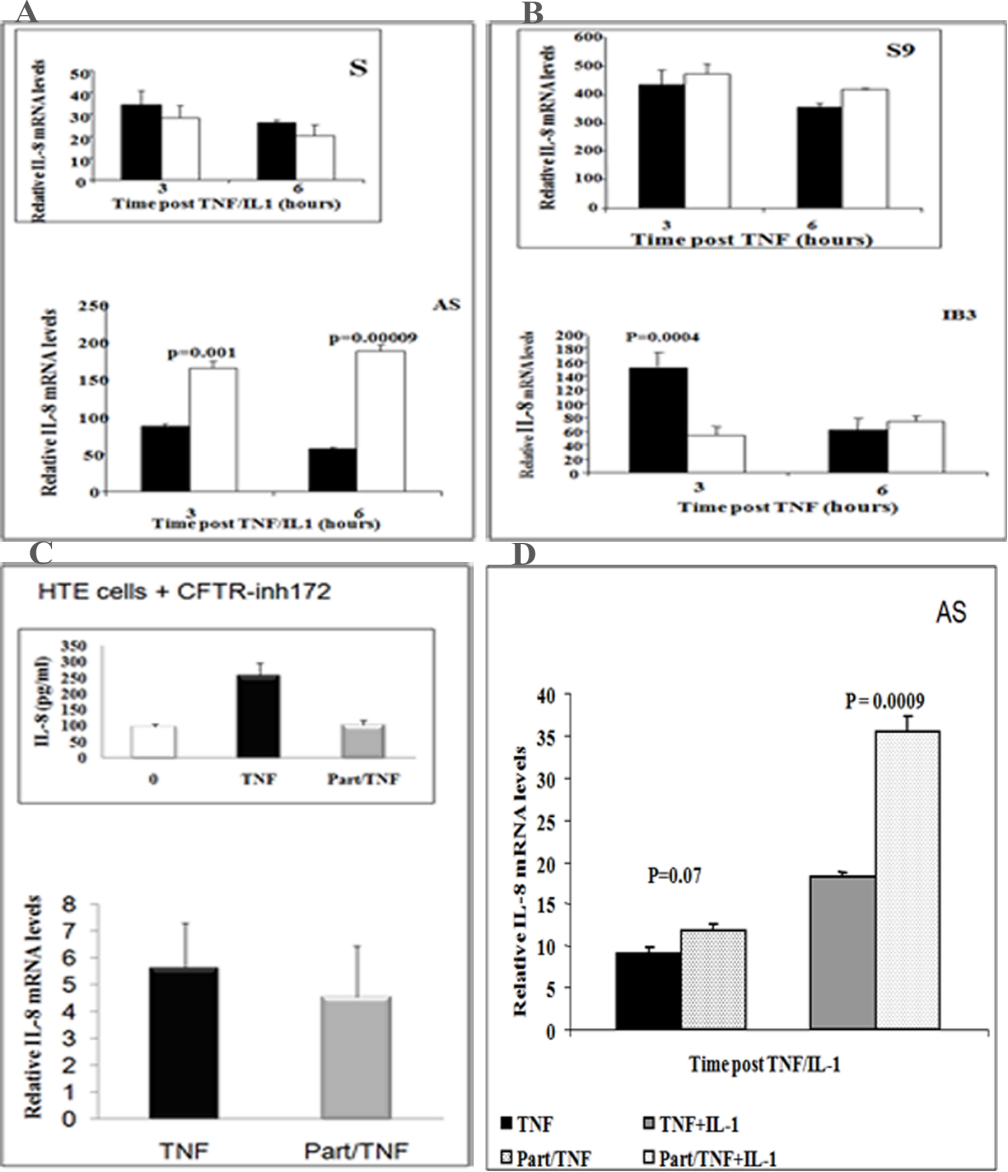
**AS/S and IB3-1/S9 cells were pretreated with parthenolide or vehicle (placebo) for 1 h and then stimulated with IL-1β and/or TNFα for the indicated time**. Subsequently, media and total RNA were prepared and analyzed for protein production and mRNA expression. The mRNA contents were normalized with for GAPDH and the results are given in relative units. (A) Parthenolide pretreatment significantly increased IL-8 mRNA expression in AS cells at 3 and 6 h (p = 0.001 and p = 0.00009, respectively) compared to S cells (inset). (B) Parthenolide pretreatment significantly inhibits IL-8 mRNA accumulation in IB3-1 cells at 3 h (p = 0.0004) compared to S9 cells (inset). (C) HTE cells were treated with 20 μM CFTR_inh_172 for 3 weeks (media changed every other day). When cells were ready, the inhibitor was added to basal and apical side and the media was replenished every day for 3 days. On the fourth day parthenolide or DMSO were added, one hour later cells were stimulated with or without TNFα for 3 h. Parthenolide significantly decreased IL-8 secretion in HTE_inh_172 at 3 h (p = 0.004), whereas, parthenolide pretreatment did not affect IL-8 mRNA accumulation (C, inset). **(D) **AS cells were pretreated with parthenolide or vehicle (placebo) for 1 h and then stimulated with TNFα alone or TNFα/IL-1β for 3 h. Subsequently, media and total RNA were prepared and analyzed for mRNA accumulation. The mRNA content was normalized with GAPDH and the results are given in relative units. Parthenolide pretreatment had no significant changes on AS-stimulated with TNFα; however it significantly increased IL-8 mRNA accumulation in AS-stimulated with TNFα/IL-1β (p = 0.0009). Results are shown as means ± SEM (n = 4-6 for each time point and condition).

Because phosphorylated p38 has been reported to stabilize IL-8 mRNA, we wanted to determine if that mechanism was operating concurrently with the inhibition of net IL-8 secretion caused by parthenolide. Therefore, we evaluated the effect of the drug on IL-8 mRNA expression as determined by RT-PCR. ELISAs were performed with supernatants from the same cells used for the real time PCR experiments, to confirm that parthenolide in fact inhibited IL-8 production during each experiment. The results verified that parthenolide inhibited IL-8 protein production in both AS and S cells (Data not shown). Parthenolide pretreatment significantly increased the content of IL-8 mRNA in TNFα/IL-1β stimulated AS at 3 and 6 h (p = 0.001 and p = 0.00009, respectively) (Figure 6A) compared to cells treated with vehicle alone (placebo, solid bars). In contrast, parthenolide treatment did not significantly increase mRNA in S cells (Figure 6A, inset). These data suggest that parthenolide treatment results in stabilization of that mRNA which is transcribed. The increased mRNA in the face of decreased protein production suggests an additional inhibitory effect at a translational level. In IB3-1 cells stimulated with TNFα alone, parthenolide pretreatment leads to the inhibition of TNFα-induced IL-8 mRNA accumulation (Figure 6B), whereas in control S9 cells it did not significantly increase IL-8 mRNA accumulation (Figure 6B, inset).

Primary HTE cells rendered "CF like" with 20 μM CFTR_inh_172 (21) were also evaluated. Figure 6C showed that IL-8 mRNA accumulation in response to TNFα alone is not significantly different between parthenolide and DMSO pretreated unstimulated HTE cells (5.6 ± 1.69 vs. 4.54 ± 1.88 respectively), whereas Parthenolide significantly inhibits the TNFα- induced increase in IL-8 secretion (p = 0.04) (Figure 6C, inset). In a separate experiment AS cells were pretreated with parthenolide, then one hour later stimulated with TNFα alone or IL-1β/TNFα together. Parthenolide significantly increased IL-8 mRNA accumulation in AS cells stimulated with IL-1β/TNFα, whereas the parthenolide effects was not observed when cells were stimulated with TNFα alone (Figure 6D). In all cases, parthenolide significantly inhibited IL-8 protein (data not shown).

### Analysis of IL-8 promoter activity after pretreatment by parthenolide and stimulation with both TNFα/IL-1β

**Figure 7:**
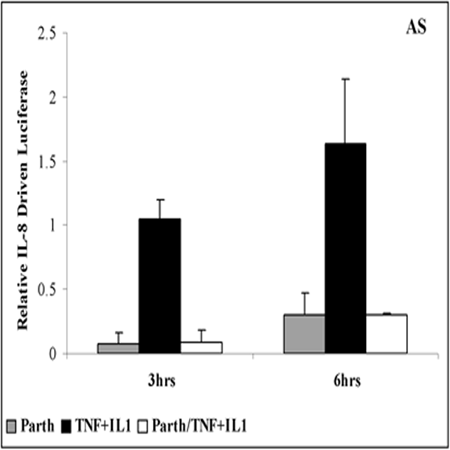
**Promoter activity after pretreatment with parthenolide and/or stimulation by TNFα/IL-1β**. AS cell lines were transfected with a plasmid-driving expression of the firefly luciferase reporter under control of IL-8 promoter. Then AS cells were pretreated with 40 μM parthenolide or vehicle for 1 h and then stimulated with TNFα/IL-1β for the 3 and 6 h. At the indicated time media was collected and subject to ELISA to determine IL-8 production. Cells were washed and then assayed for luciferase activity. Firefly luciferase activity was normalized against a constitutively expressed *Renilla *luciferase reporter. Results are expressed as relative luciferase activity above control cells transfected and pretreated with DMSO.

Because the observation of increased IL-8 mRNA in AS cells seems paradoxical in the face of inhibition of activation of the transcription factors AP-1 and NFκB, which are considered the most important regulators of IL-8 gene expression, we sought to directly investigate the influence of parthenolide on transcription independently of possible effects on IL-8 mRNA stability or translation. AS and S cell lines were transfected with a plasmid driving expression of the firefly luciferase reporter under control of the IL-8 promoter [23]. Firefly luciferase activity was normalized against a constitutively expressed *Renilla *luciferase reporter. AS and S cell lines were pretreated with parthenolide or DMSO, then one hour later stimulated with TNFα/IL-1β for 3 h. Supernatants were collected and assessed by ELISA for IL-8 production and the cells were assessed for IL-8 promoter activity. IL-8 promoter activity responded to TNFα/IL-1β with 1.5-2 fold increases in luciferase expression at 3 and 6 hrs respectively in AS cells (Figure 7). Parthenolide inhibited this stimulated firefly luciferase expression as well as the basal expression without stimulation (Figure 7), confirming inhibition of transcription. ELISAs verified that parthenolide inhibited the production of IL-8 protein in response to TNFα/IL-1β (data not shown).

## Discussion

Human bronchial epithelial cell lines with defective CFTR expression and/or function have exaggerated IL-8 mRNA expression and protein secretion in response to stimulation with TNF-α and IL-1β [7]. This result is in agreement with most other studies of IL-8 mRNA in CF cells and tissues [13,18,28,29]. We and others have previously shown that this is associated with increased and prolonged activation of IKK and NFκB in CF as compared to non-CF cells [10,13,14,30]. The present results confirm that inflammatory cytokine stimulation causes a huge increase in IL-8 production and gene expression in CF compared with control cells. Furthermore, we show that the increased IL-8 production is also associated with: 1) excessive and prolonged activation of AP-1 in addition to NFκB, and 2) excessive and prolonged activation of the MAP Kinases p38, JNK and ERK. Thus, the MAPKs/AP-1 signaling pathways as well as the IKK/NFκB pathway shows exaggerated and prolonged activation after stimulation of cells with CF defects. Both pathways likely contribute to the excessive amount of IL-8 mRNA and excessive protein secretion which characterizes CF.

IL-8 is a potent chemokine which attracts neutrophils to the lung. An excess of this chemokine is believed to make an important contribution to the excessive influx of neutrophils that ultimately causes lung damage and death in CF patients. A large body of evidence has shown that the increased IL-8 secretion in CF is associated with excessive activation of IKK and NFκB [10,12-14,30]. However, little is known about IL-8 regulation in the context of mitogen-activated protein kinases (MAPKs) and the transcription factor, activator protein-1 (AP-1) in CF. Three MAPK pathways are believed to contribute to IL-8 gene expression, the extracellular-regulated protein kinase (ERK), JUN-N-terminal protein kinase (JNK), and p38 MAPK [21]. To our knowledge there are few studies that report a higher ERK activation in CF cells [15], and no study about the MAPK p38 or the transcription factor AP-1. Blau and colleagues however did not demonstrate a prolonged activation of ERK, which we believe to be of major interest in CF disease. We believe that our paper for the first time showed that all 3 MAPKs and AP-1 are dysregulated and their activation is excessive and prolonged in CF. All of these are activated by phosphorylation through dual-specificity MAP kinase kinases, also referred to as MKK [16,17,31]. Holtmann and colleagues found that introduction of an activating mutation in MKK6, increased p38 and, stabilized IL-8 mRNA and further increased IL-8 protein formation induced by NFκB-inducing kinase (NIK) [16]. The active form of the MAPK-activated protein kinase 2 (MK-2), a downstream substrate of the p38 MAPK pathway, also induced mRNA stabilization. Negative mutants of MK-2 decreased mRNA stability [16,32]. This data suggest that IL-1β and TNFα can induce IL-8 production through simultaneous activation of MAPK cascades that regulate AP-1 in addition to the IKK pathway, which activates NFκB [17]. Unlike NFκB, AP-1 is not essential for transcription of IL-8 but it is required for maximal gene expression [16,17,24]. Further cooperation of these two pathways resulting in maximal IL-8 secretion may be due to activation of IKK by MAPK kinase kinase (also called MEKK1), which also activates the three MAPKs [29]. In addition, NFκB and AP-1 modulate each other and can function cooperatively [18,29,28,33] for rapid and maximum transcriptional activation. However, their activities are also rapidly down-regulated in normal cells so that rigorous responses are often transient.

Our results clearly show that in epithelial cells with CF defect, stimulation with TNFα alone or TNFα/IL-1β results in marked increases in the amounts of phospho-p38, phospho-ERK, and phospho-JNK p46/p54 as compared to control cells. The time intervals at which the phosphorylated intermediates of the different MAPKs differ from each other, but for all three, phosphorylated forms are present for longer intervals after stimulation in "CF-Like" cells than in similarly stimulated "Wild-Type-Like"cells. These results correlate with, and likely account for the increased AP-1 observed at all time intervals after stimulation in CF cells as compared to non-CF cells. Thus, the CF defect seems to have similar effects on the MAPK/AP-1 pathway as on the IKK/NFκB pathway. The persistent phosphorylation of all 3 MAPKs; ERK, JNK and p38 in cells with defective CFTR, suggest that CF alters a phosphatase whose decreased activity could explain the persistence of these enzymes. Together, simultaneous and prolonged activation of these two transcription factors is very likely to play a major role in the excessive and prolonged IL-8 production seen in CF cells and patients.

Parthenolide, a sesquiterpene lactone found in the medicinal plant feverfew (*Tanacetum parthenium*) has powerful anti-inflammatory properties [14,34,35]. We have previously showed that parthenolide inhibits inflammation in CF both in vivo and in vitro by inhibiting IKK/NFκB pathway. Parthenolide has been considered by some to be a specific inhibitor of the NFκB pathway [34-39]. During these studies, we wished to determine the effects of this inhibitor on the MAPKs and AP-1 pathway, because of the similar reactions and likely cross-talk between the two pathways which lead to formation of AP-1 and activation of NFκB, respectively. In agreement with previous results, we found that parthenolide inhibited secretion of IL-8 protein by CF as well as non-CF cells. Our new results show that parthenolide inhibited ERK phosphorylation induced by TNFα/IL-1β stimulation, and also inhibited AP-1 activation; but that phosphorylated p38 was preserved by this inhibitor. Since phosphorylated p38 can play an important role in stabilization of the mRNAs for pro-inflammatory cytokines, we sought to dissect the possible effects of parthenolide on transcriptional activation of the IL-8 promoter versus its net effects on IL-8 mRNA in cells stimulated with TNFα/IL-1β. Results using a luciferase reporter construct show that parthenolide inhibited transcription per se. However, using real time PCR, we found that parthenolide pretreatment, actually increased IL-8 mRNA in TNFα/IL-1β stimulated cells. This is most likely a consequence of the preservation of phosphorylated p38 caused by the drug [16,32]. since phospho-p38 is known to play an important role in modulating the effect of the AU rich sequences that would otherwise destabilize pro-inflammatory cytokine mRNAs. In turn, the preservation of phospho-p38 in the face of decreased phosphorylated ERK suggests the possibility that parthenolide acts on a phosphatase which specifically targets p38 [40,41]. The results also suggest that parthenolide inhibits translation of the IL-8 message, accounting for the apparent paradox of finding increased mRNA in the face of decreased protein secretion.

The differences between cell lines is probably due to the sources, however across each model parthenolide pretreatment results in an inhibition of inflammatory signaling in cells with CF defects.

Production of IL-8 and other pro-inflammatory cytokines is also governed by mechanisms regulating in their mRNA half-lives. Several reports have shown that IL-8 mRNA degradation is modulated by AU-rich sequences present in the 3'UTR of IL-8 mRNA [23,28,42]. These sequences function as potent destabilizing elements that cause rapid decay of the transcripts. Furthermore, a recently identified family of genes called micro-inhibitory RNAs (miRNAs) has been shown to regulate protein expression mainly at the post-transcriptional level [43], and has been shown to be dysregulated in several inflammatory diseases including psoriasis, rheumatoid arthritis and asthma [44,45]. The miRNA/mRNA interaction occurs within the 3'UTR of the target mRNA and decreases protein translation. Since our results suggest that parthenolide also inhibits translation (see below), we speculate that this may involve stabilization of these inhibitory miRNAs, which in turn inhibits IL-8 mRNA translation. This hypothesis would best explain our results, which showed decreased IL-8 protein secretion despite increased message concomitantly with stabilization of phospho-p38.

Overall, the results in three different cellular models of CF show remarkable similarity in the ways that multiple different pathways of transcription factor signaling are affected by the CF defect. In response to physiologic stimulation, phosphorylation and activation of multiple MAPKs is greater and persists for longer in CF airway epithelial cells as compared to non-CF airway epithelial cells. The termination of the activities of MAPKs and IKK is normally insured by dual specificity phosphatases (DUSP), also called MAP kinase phosphatase (MKP) [46-48].

Our data showing increased activity of multiple kinase pathways may therefore suggest that decreased expression or function of CFTR somehow decreases activity of a multi-specific protein phosphatase [49]. Although this particular study has focused only on one important pro-inflammatory cytokine, IL-8, it is likely that many other genes are also over-expressed in CF as a consequence of the dysregulation of these signaling pathways.

## Conclusions

Our results suggest that 1) in addition to IKK/NFκB pathway, MAPKs and AP-1 are also dysregulated in CF epithelial cells, and 2) the effects of a widely used inhibitor, parthenolide, are less specific than previously believed. However, the results also suggest that this type of compound, which simultaneously targets multiple signaling pathways, may have beneficial effects in CF and other diseases in which multiple mechanisms that should control inflammation are disrupted.

## Abbreviations

CF: Cystic fibrosis; MAPKs: Mitogen activated protein kinases; HBEC: Human bronchial epithelial cells; ERK: Extracellular-regulated protein kinase; JNK: Jun-N terminal protein kinase; AP-1: Activator-protein-1; 16 HBE: human bronchial epithelial cell line; AS: 16HBE stably transfected with antisense oligonucleotides which inhibit expression of CFTR; IB3-1: Genetically CF Clinical sample; CFTR-Inhibitor 172: CFTR_inh_-172

## Competing interests

The authors declare that they have no competing interests.

## Authors' contributions

MB and AS participated in the conception and design of the study, TLB contributed to the design and process of the review in the later phases of experimentation. MB, TLB and EJ reviewed the article for important intellectual content and clarity. AS conducted the literature search, acquisition of data analysis, interpretation of data, wrote the first draft of the manuscript and composed and edited the figures. EJ performed the luciferase assay experiments. All authors read and approved the final manuscript.
